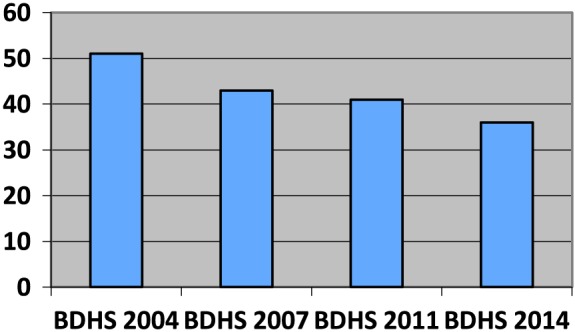# Imperatives for reducing child stunting in Bangladesh†

**DOI:** 10.1111/mcn.12284

**Published:** 2016-05-17

**Authors:** Tahmeed Ahmed, Muttaquina Hossain, Mustafa Mahfuz, Nuzhat Choudhury, Shamim Ahmed

**Affiliations:** ^1^ Nutrition and Clinical Services Division Dhaka Bangladesh; ^2^ WaterAid Dhaka Bangladesh

Although there has been a decline in rates of child stunting in Bangladesh, more than one‐third of under‐five children still suffer from impaired linear growth. Results of the demographic health surveys since 2004 show that the rate of stunting decreased by only 1.5 percentage points per year (NIPORT, Mitra and Associates & ICF International, 2015). This is not anticipated, given the impressive results the country has demonstrated in reducing infant and maternal mortality over the past two decades (NIPORT, MEASURE Evaluation, and ICDDR,B 2012). Stunting seems to be pervasive throughout the country and is very high in children in slum settlements. Poverty and lack of education are associated with stunting in Bangladesh as elsewhere; however, 21% children from households belonging to the richest wealth quintiles are also stunted. About one‐third of children of literate mothers also suffer from stunting. Therefore, the aetiology of stunting is still not clear, although data from Bangladesh suggest that factors associated with the condition include poor maternal nutrition, low birthweight (LBW), severe food insecurity, inappropriate complementary feeding, poverty, illiteracy, poor sanitation, and hygiene practices (Ahmed *et al*, 2012; Psaki *et al*., 2014). There is increasing evidence that environmental enteropathy, a condition where the small intestinal mucosa is colonized and damaged by pathogenic bacteria, is one cause of malabsorption of nutrients and stunting. This happens when hygiene and sanitation practices are poor and young children are chronically exposed to bacteria in the environment.

The pace at which stunting reduction is taking place in Bangladesh is not enough to achieve the World Health Assembly target of 40% reduction in stunting levels by 2025 (IFPRI, 2014). The current annual average rate of reduction is 2.7%, and this will need to be increased to 3.3% to achieve the target. This is not a phenomenal increase compared with the current rate of reduction, but it will require concerted planning and efforts. From an economic perspective, the cost to Bangladesh of not investing in accelerating this reduction will be huge in terms of lost gross domestic product and income. From a health perspective, it is essential to improve linear growth of children so that the negative effects of excess ponderal growth and resulting overweight and obesity are attenuated.

Priority action areas for improving linear growth and increasing the decline in rate of stunting in Bangladesh are summarized in the succeeding text.

## Effective multisectoral coordination of nutrition interventions

This is essential for implementation of nutrition‐specific and sensitive interventions at all levels. Given the extent and magnitude of the nutritional problems in the country, it is highly desirable that the national level multi‐sectoral coordination be directed from the Prime Minister's office. Experience in other countries suggests that improving public health nutrition has been successful where the highest level of government office was involved in giving high level directives for coordinating policy and national level implementation.

## Formulating a national plan of action for nutrition

The government has recently approved the new national nutrition policy, which is based on new evidence. It is now important to have a realistic national plan of action and link financing for this plan, in line with the policy. It should include an assessment of health‐related human resources, particularly at the grass‐roots level, for effective behaviour change communication. It should also have a robust mechanism for monitoring and feedback for course correction of implementation, as well as accountability mechanisms at all levels.

## Scaling up nutrition‐specific and nutrition‐sensitive interventions

Nutrition‐specific interventions include balanced energy‐protein supplementation and iron‐folic acid, calcium supplementation during pregnancy; counselling to support breastfeeding and appropriate complementary feeding, micronutrient supplementation to young children, and treatment of moderate and severe acute malnutrition. These are essential nutrition interventions, but scaling them up can reduce mortality by 15% and stunting by only 20% (Bhutta *et al*., 2013). This implies that these have to be combined with nutrition‐sensitive interventions, which can assure adequate water, sanitation and hygiene, food security, family planning, improving literacy, social safety net programmes and poverty alleviation. Economic and social policies that increase minimum wages and reduce socioeconomic inequalities should be formulated and implemented.

## Reduction in low birthweight

Bangladesh has one of the highest rates of LBW in the world (36%) (UNICEF and Bangladesh Bureau of Statistics, 2005). One‐fifth of stunting is attributed to LBW. Therefore, the importance of reducing LBW cannot be overemphasized. This can be attempted through a life cycle approach by improving health and nutrition of adolescent girls and encouraging optimum family food, rest, and proper antenatal care during pregnancy. Iron‐folic acid supplementation during pregnancy has a beneficial effect on birthweight and its coverage should be increased. Recent evidence suggests multiple micronutrient supplementations might have an even greater impact (West *et al*., 2014).

## Nutrition education in secondary school (grade 6 to grade 10) curriculum

The current focus of nutrition education in school curricula is very limited. Educational curricula should emphasize the importance of appropriate nutrition for adolescent girls, pregnant and lactating mothers, and breastfeeding and complementary feeding of young infants as routes to better linear growth in Bangladesh.

## Water, sanitation and hygiene

Although rates of open defecation have decreased to about 4% in Bangladesh, some critical hygiene and sanitation problems remain. These include proper disposal of excreta; clogged drainage systems and garbage in urban areas; handwashing at critical times; and safe preparation and storage of complementary food. Communications campaigns and further investment in waste management to reduce environmental enteropathy, a potential cause of stunting, can help greatly.

## Population control

With a small land mass, Bangladesh has the highest population density in the world. The fertility rate has declined, but it could be brought below replacement levels by rejuvenating the family planning programme and scaling up both temporary and permanent methods of contraception in an inclusive manner.

## Poverty alleviation

Although stunting is not restricted to the poor, poverty is a major constraint to ensuring nutritious diets and adequate health care. It also leads to migration rural‐urban migration or poor city areas where the situation is often worse. Currently, 21% of the population is extremely poor (Sen & Ali, 2015); rapid and effective implementation of the government's new social protection strategy and an emphasis on poverty alleviation in the upcoming seventh 5‐year plan for the country are essential.

## Food security

While food insecurity, as assessed for its access to food domain, has declined, it still affects 32% of the population (Helen Keller International and James P Grant School of Public Health, 2013). Opportunities to address food insecurity include improving livelihoods and income and increasing awareness regarding healthy diets and food supplementation where needed, that is, hard to reach or extremely food insecure conditions.

## Literacy

Low maternal education and low literacy are key determinants of stunting. National efforts to bring and retain girls in schools and colleges need to be intensified.

Bangladesh is clearly a leader in reducing stunting across the South Asia region (Headey *et al*., 2014), but the levels of stunting still remain too high. For Bangladesh, and for the region, such high rates of stunting are unacceptable. We call for the government, development partners, researchers, and Bangladeshi society to act together to improve the various known drivers of stunting reduction in Bangladesh.

## Source of funding

This research activity was funded by core donors to icddr,b including: Government of the People's Republic of Bangladesh; the Department of Foreign Affairs,Trade and Development (DFATD), Canada; Swedish International Development Cooperation Agency (Sida) and the Department for International Development (UKAid). We gratefully acknowledge these donors for their support and commitment to icddr,b's research efforts.

## Conflicts of interest

The authors declare that they have no conflicts of interest.

## Contributions

TA conceptualized and wrote the first draft of the paper. MH, MM, NC and SA substantially contributed by revising the paper.

## Prevalence of stunting among under‐five children in Bangladesh: results of demographic health surveys, 2004–2014